# Aldioxa improves delayed gastric emptying and impaired gastric compliance, pathophysiologic mechanisms of functional dyspepsia

**DOI:** 10.1038/srep17519

**Published:** 2015-12-01

**Authors:** Teita Asano, Shuji Aida, Shintaro Suemasu, Kayoko Tahara, Ken-ichiro Tanaka, Tohru Mizushima

**Affiliations:** 1 Department of Drug Discovery and Development, Faculty of Pharmacy, Keio University, 1-5-30, Shibakoen, Minato-ku, Tokyo 105-8512, Japan; 2 LTT Bio-Pharma Co., Ltd, Shiodome Building 3F, 1-2-20 Kaigan, Minato-ku, Tokyo 105-0022, Japan

## Abstract

Delayed gastric emptying and impaired gastric accommodation (decreased gastric compliance) play important roles in functional dyspepsia (FD). Here we screen for a clinically used drug with an ability to improve delayed gastric emptying in rats. Oral administration of aldioxa (dihydroxyaluminum allantoinate) partially improved clonidine- or restraint stress-induced delayed gastric emptying. Administration of allantoin, but not aluminium hydroxide, restored the gastric emptying. Both aldioxa and allantoin inhibited clonidine binding to the α-2 adrenergic receptor, suggesting that antagonistic activity of the allantoin moiety of aldioxa on this receptor is involved in the restoration of gastric emptying activity. Aldioxa or aluminium hydroxide but not allantoin restored gastric compliance with restraint stress, suggesting that aluminium hydroxide moiety is involved in this restoration. We propose that aldioxa is a candidate drug for FD, because its safety in humans has already been confirmed and its ameliorating effect on both of delayed gastric emptying and impaired gastric compliance are confirmed here.

Functional dyspepsia (FD) is a disease defined as persistent or recurrent postprandial upperabdominal discomfort and epigastric pain in the absence of any organic, systemic, or metabolic diseases. The prevalence of dyspepsia in the general population is remarkably high (15–20%) and the majority of these patients is believed to have FD^1^. Although FD is not a life-threatening disease, it markedly reduces patients’ quality of life and places a significant economic burden on the healthcare system^2^. However, protocols to treat FD, including pharmacotherapy, have not been fully efficacious, with approximately 50% of FD patients remaining symptomatic over a 5-year follow-up period^1^.

According to Rome III criteria, FD is categorized into postprandial-distress syndrome (PDS) and epigastric pain syndrome (EPS), based on the predominant symptoms (postprandial fullness/early satiation and epigastric pain/burning, respectively)^3^,^4^. Delayed gastric emptying and impaired gastric accommodation to a meal or visceral hypersensitivity to gastric distension have been suggested to be involved in the pathogenesis of PDS or EPS, respectively^5^.

While the mechanism of delayed gastric emptying in FD patients has been widely investigated over the past two decades, no single definitive reason for the condition has been identified. Thus, a combination of physiologic, genetic, environmental and psychological factors are supposed to cause delayed gastric emptying seen in FD patients. Various endogenous molecules, including hypothalamic-pituitary-adrenal axis system-related molecules (such as corticotropin-releasing hormone and corticosterone), ghrelin, serotonin (5-HT) and dopamine affect gastric emptying and were suggested to be involved in delayed gastric emptying in FD patients^6^,^7^. Consequently, these molecules and their receptors have received considerable attention as targets for anti-FD drugs. For example, mosapride citrate (mosapride), cisapride monohydrate (cisapride) and tegaserod are 5-HT_4_ receptor agonists, while domperidone and itopride hydrochloride (itopride) are dopamine 2 (D_2_) receptor antagonists; all of these drugs are currently used or had been used to treat FD patients in some countries^8^. However, pharmacological therapy by these drugs for FD patients has resulted in outcomes that have been unsatisfactory^9^. Furthermore, since these endogenous molecules also regulate other physiological functions, especially heart function, the use of these drugs is restricted clinically due to adverse effects, such as cardiotoxicity. In fact, cisapride has been withdrawn from the US market due to its cardiotoxic properties (long QT syndrome)^10^. Thus, new target molecules for anti-FD drugs need be identified, one approach to follow is the phenotype screening of compounds that could stimulate gastric emptying in animals.

Acetylcholine is also a key positive regulator for gastric emptying and it was reported that dysfunction of the vagus nerve and a resulting decreased level of acetylcholine release from nerve terminals are involved in the delayed gastric emptying seen in FD patients^11^,^12^. Furthermore, acotiamide hydrochloride (acotiamide), the first drug approved to treat FD in Japan, is an acetylcholinesterase inhibitor and was suggested to stimulate gastric emptying by increasing acetylcholine levels in vagus nerve terminals^13^. Various receptor types, such as the α-2 adrenergic, D_2_ and 5-HT_4_ receptors, affect the gastric emptying, at least partially, through regulation of this acetylcholine release^14^,^15^. Thus, drugs that affect the acetylcholine release might be good candidates as anti-FD drugs.

Gastric accommodation provides temporary storage for ingested food prior to its transition into the intestine. This process consists of a reduction in gastric tone and an increase in compliance in response to food intake, allowing an increased fundic volume without any rise in intragastric pressure^16^. In addition to delayed gastric emptying, impaired gastric accommodation plays an important role in the pathogenesis of FD, particularly PDS^5^,^17^. The importance of impaired gastric accommodation in the pathogenesis of FD was further suggested by a recent finding that acotiamide stimulates gastric accommodation^18^, although the mechanism is unknown.

The number of drugs reaching the marketplace each year is decreasing, mainly due to the unexpected adverse effects of potential drugs being revealed in clinical trials. Thus, we have proposed a new strategy for drug discovery and development (drug re-positioning strategy), which focuses on the use of existing medicines for alternative indications^19^. In this strategy, compounds with clinically beneficial pharmacological activity are screened from a library of medicines already in clinical use to be developed for new indications. The advantage of this strategy is that there is a decreased risk for unexpected adverse effects in humans because the safety aspects of these drugs have already been well characterized^19^.

In the present study, we undertook both strategies of phenotypic screening in animals and drug re-positioning to search candidate drugs for FD. The drug aldioxa, a medicine used clinically for many years to treat gastric ulcers and gastritis, was identified^20^,^21^. Orally administered aldioxa partially restored gastric emptying that was impaired by the administration of clonidine hydrochloride (clonidine) or in response to restraint stress. Our results suggest that aldioxa restores delayed gastric emptying via its capacity to antagonise α-2 adrenergic receptor activity. We also found that aldioxa could increase gastric compliance in rats subjected to wrap restraint stress. Based on these results, we propose that aldioxa could serve as a candidate drug for FD given that its safety in humans has previously been confirmed.

## Results

### Effect of aldioxa on clonidine-induced delayed gastric emptying

From a group of 209 drugs already in clinical use, we screened for compounds able to suppress clonidine-induced delayed gastric emptying; each drug was administered intraperitoneally to ICR mice at a dose 30 times higher than its clinical dose, then clonidine was administered and the gastric emptying was measured by the phenol red marker method. In this manner, we selected aldioxa based on the level of restoration of gastric emptying and on clinical data attesting to its efficacy and safety. Aldioxa has been used clinically in Japan for many years to treat gastric ulcers and gastritis^20^,^21^.

**Figure 1:**
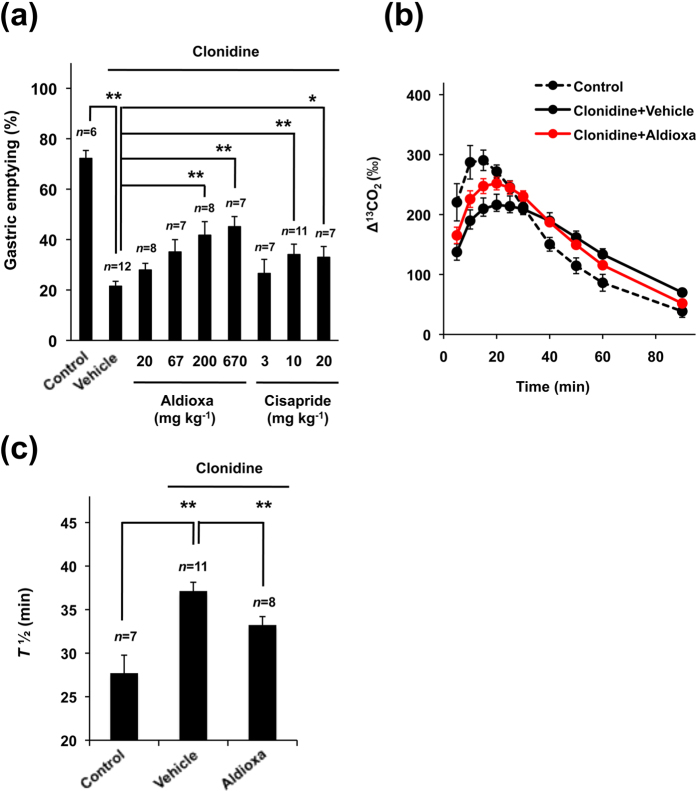
Effect of aldioxa on clonidine-induced delayed gastric emptying. Mice fasted for 18 h were orally administered indicated dose (**a**) or 200 mg kg^−1^ (**b**,**c**) of aldioxa, indicated dose of cisapride (**a**) or vehicle (1% methylcellulose) (**a**–**c**). Fifty-five (**a**) or forty-five (**b**,**c**) minutes after each drug administration, delayed gastric emptying was induced by the subcutaneous administration of clonidine (100 (**a**) or 30 (**b**,**c**) μg kg^−1^). Control mice were subcutaneously administered saline (**a**–**c**). Gastric emptying was measured using the phenol red method (**a**) or the [^13^C]-labeled acetic acid breath test (**b**,**c**) and gastric emptying (**a**), Δ^13^CO_2_ (%) (**b**) and *T*_1/2_ (**c**) were calculated as described in the Methods. Values are mean ± s.e.m. *P < 0.05; **P < 0.01 (Tukey test). Experiments were replicated at least two times.

To begin with, we examined the effect of oral administration of aldioxa on clonidine-induced delayed gastric emptying. As shown in Fig. 1a, administration of clonidine delayed gastric emptying, and oral pre-administration of aldioxa partially restored the gastric emptying in a dose-dependent manner. The significant restoration was observed at both 200 and 670 mg kg^−1^ and the extent of restoration was similar between these doses (Fig. 1a). Restorative activity was also observed in mice treated with cisapride (Fig. 1a) as described previously^22^.

Gastric emptying in mice was also measured by the [^13^C]-labeled acetic acid breath test. Orally administered [^13^C]-labeled acetic acid is rapidly absorbed in the small intestine (but not in the stomach) and metabolized to ^13^CO_2_. Thus, by monitoring the time course ^13^CO_2_ content in the expired air, we can evaluate gastric emptying^23^. As shown in Fig. 1b, administration of clonidine decreased the peak level of Δ^13^CO_2_ (%; relative amount of ^13^CO_2_ in expired air) and delayed the time to peak, showing that clonidine delays gastric emptying. Pre-administration of aldioxa returned the Δ^13^CO_2_ (%) profile towards that of control mice (without clonidine treatment) (Fig. 1b), suggesting that aldioxa partially improves clonidine-induced delayed gastric emptying. Gastric emptying ability can be estimated from *T*_1/2_ of the *AUC*_90 min_ of Δ^13^CO_2_ (%) (time when half of *AUC*_90 min_ of Δ^13^CO_2_ (%) had been reached)^23^. As shown in Fig. 1c, administration of clonidine prolonged the *T*_1/2_ value, which was partially returned to control levels by pre-administration of animals with aldioxa.

### Relationship between gastroprotective effect and ameliorating effect on delayed gastric emptying of aldioxa

**Figure 2:**
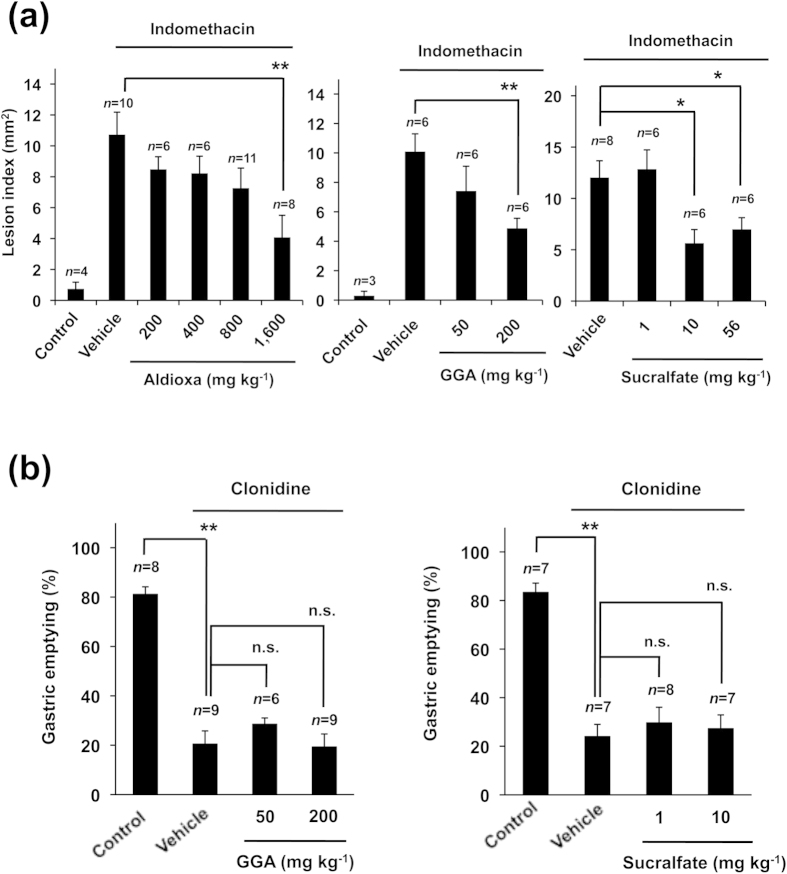
Effect of gastroprotective drugs on clonidine-induced delayed gastric emptying. Mice fasted for 18 h were orally administered indicated dose of aldioxa, geranylgeranylacetone (GGA) or sucralfate. One hour later, 20 mg kg^−1^ of indomethacin was orally administered and 8 h later, stomachs were removed and gastric mucosal lesions were measured (**a**). The effect of GGA or sucralfate at indicated dose on clonidine-induced delayed gastric emptying was examined using the phenol red method as described in the legend of Fig. 1 (**b**). Values are mean ± s.e.m. *P < 0.05; **P < 0.01 (Tukey test). n.s., not significant. Experiments were replicated at least two times.

Aldioxa is the generic name for the metal complex, dihydroxyaluminum allantoinate, which is hydrolyzed to allantoin and aluminium hydrate at the gastric mucosa^24^,^25^. The molecular mechanism governing the gastroprotective effect of aldioxa is unclear; however, it was suggested that both the direct coating of the gastric mucosa with aluminium hydrate and the anti-inflammatory effect of allantoin are involved^26^,^27^. To understand the relationship between aldioxa’s gastroprotective effect and its stimulatory effect on gastric emptying, we compared its dose-response profiles on these effects. As shown in Fig. 2a, compared to its actions on delayed gastric emptying (Fig. 1a), a much higher dose of aldioxa (1,600 mg kg^−1^) was required to suppress indomethacin-induced gastric lesions. We also examined the effects of other gastroprotective drugs on clonidine-induced delayed gastric emptying. Neither geranylgeranylacetone (GGA) nor sucralfate affected clonidine-induced delayed gastric emptying (Fig. 2b) at doses that could suppress indomethacin-induced gastric lesions (Fig. 2a).

**Figure 3:**
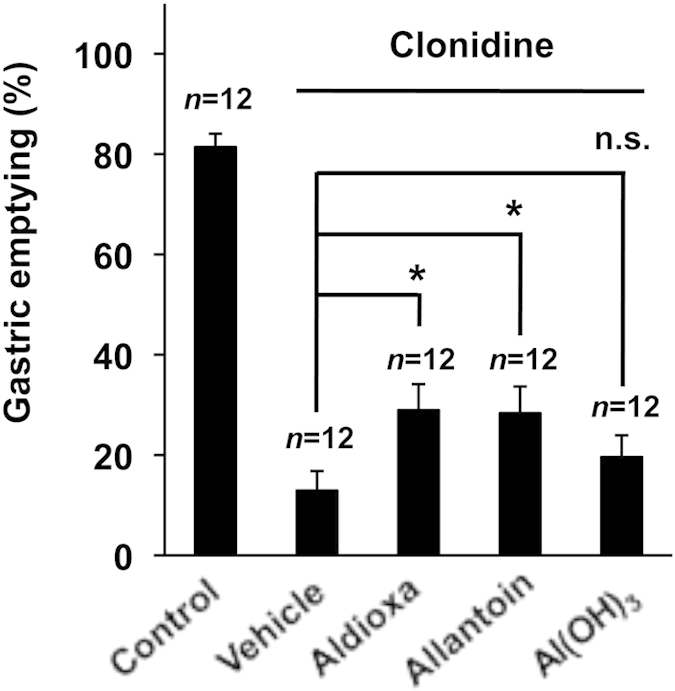
Effect of allantoin or aluminium hydrate on clonidine-induced delayed gastric emptying. Mice fasted for 18 h were orally administered aldioxa (200 mg kg^−1^), allantoin (145 mg kg^−1^), aluminium hydrate (Al(OH)_3_) (72 mg kg^−1^) or vehicle (1% methylcellulose). Clonidine-induced delayed gastric emptying was induced and measured using the phenol red method as described in the legend of Fig. 1. Values are mean ± s.e.m. *P < 0.05 (Tukey test). n.s., not significant. Experiments were replicated at least two times.

We also separately examined the effect of allantoin or aluminium hydrate on clonidine-induced delayed gastric emptying. As shown in Fig. 3, allantoin but not aluminium hydrate partially improved clonidine-induced delayed gastric emptying at an equivalent dose to that of aldioxa (with respect to molecular number, 200 mg kg^−1^ of aldioxa corresponds to 145 or 72 mg kg^−1^ of allantoin or aluminium hydrate, respectively). The results in Figs 2 and 3 thus suggest that aldioxa achieves its stimulatory effect on gastric emptying independently of its gastroprotective effect, and that the allantoin moiety of aldioxa is important for improved emptying.

### Effect of aldioxa on restraint stress-induced delayed gastric emptying and normal gastric emptying

**Figure 4:**
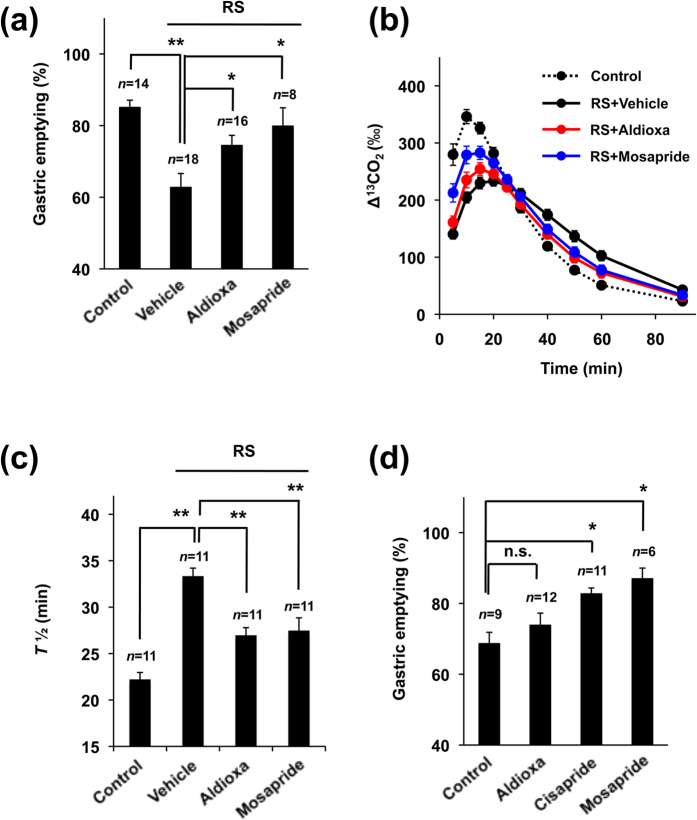
Effect of aldioxa on restraint stress-induced delayed gastric emptying and normal gastric emptying. Mice fasted for 18 h were orally administered aldioxa (200 mg kg^−1^) (**a**–**d**), mosapride (7.7 mg kg^−1^) (**a**–**d**), cisapride (5 mg kg^−1^) (**d**) or vehicle (1% methylcellulose) (**a**–**d**) and delayed gastric emptying was induced by restraint stress (RS) (**a**–**c**). One hour after the administration of each test compound, gastric emptying was measured using the phenol red method (**a**,**d**) or the [^13^C]-labeled acetic acid breath test (**b**,**c**) as described in the legend of Fig. 1. Values are mean ± s.e.m. *P < 0.05; **P < 0.01 (Tukey test). n.s., not significant. Experiments were replicated at least two times.

Restraint stress-induced delayed gastric emptying has been used as an alternative animal model of FD^28^, prompting us to examine here the efficacy of aldioxa in this model. As shown in Fig. 4a, the phenol red method revealed that mice subjected to restraint stress showed delayed gastric emptying, which was partially suppressed by the pre-administration of aldioxa or mosapride to animals. Similar results were observed with the [^13^C]-labeled acetic acid breath test (Fig. 4b,c).

We also examined the effect of orally administered aldioxa on the basal level of gastric emptying in intact mice (without restraint stress or clonidine treatment). Administration of cisapride or mosapride accelerated normal gastric emptying in intact mice as described previously^28^,^29^. However, aldioxa did not affect normal gastric emptying (Fig. 4d).

### Mechanism for the stimulatory effect of aldioxa on gastric emptying

**Figure 5:**
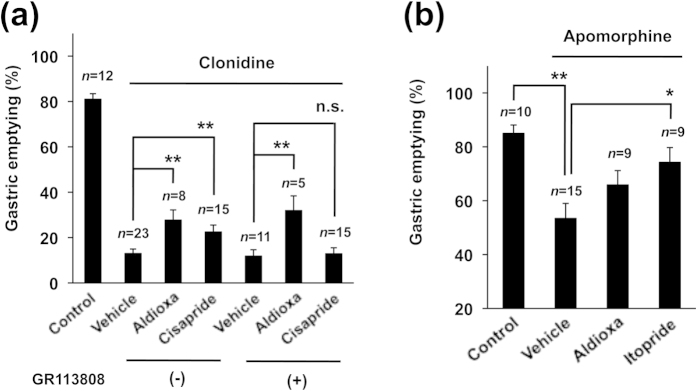
Stimulatory effect of aldioxa on gastric emptying was independent of 5-HT_4_ and D_2_ receptors. Mice fasted for 18 h were orally administered aldioxa (200 mg kg^−1^) (**a**,**b**), cisapride (5 mg kg^−1^) (**a**), itopride (200 mg kg^−1^) (**b**) or vehicle (1% methylcellulose) (**a**,**b**). GR113808 (10 mg kg^−1^), a selective 5-HT_4_ antagonist, was administered 5 min before each drug administration (**a**). Forty-five (**a**) or thirty (**b**) minutes after each drug administration, delayed gastric emptying was induced by administration of clonidine (100 μg kg^−1^) (**a**) or apomorphine (5 mg kg^−1^) (**b**) and examined using the phenol red method as described in the legend of Fig. 1. Values are mean ± s.e.m. *P < 0.05; **P < 0.01 (Tukey test). n.s., not significant. Experiments were replicated at least two times.

As described in the introduction, a number of molecular targets of drugs that could restore gastric emptying have been proposed, particularly those involving the α-2 adrenergic, D_2_ and 5-HT_4_ receptor types. We examined here the involvement of these receptors in the stimulatory effect of aldioxa on gastric emptying. To test the involvement of the 5-HT_4_ receptor, we examined the effect of a selective 5-HT_4_ receptor antagonist (GR113808)^30^ on the stimulatory effect of aldioxa on gastric emptying. As shown in Fig. 5a, aldioxa suppressed clonidine-induced delayed gastric emptying even in the presence of GR113808. On the other hand, cisapride (a 5-HT_4_ agonist) did not affect clonidine-induced delayed gastric emptying in the presence of GR113808 (Fig. 5a).

To test the involvement of the D_2_ receptor, we next examined the effect of aldioxa on (*R*)-apomorphine hydrochloride hemihydrate (apomorphine, a dopamine receptor agonist)-induced delayed gastric emptying. As shown in Fig. 5b, aldioxa had no effect on this treatment. On the other hand, administration of itopride (a D_2_ receptor antagonist) suppressed the apomorphine-induced delayed gastric emptying (Fig. 5b). These results suggest that aldioxa restores gastric emptying independently of D_2_ and 5-HT_4_ receptor involvement.

**Figure 6:**
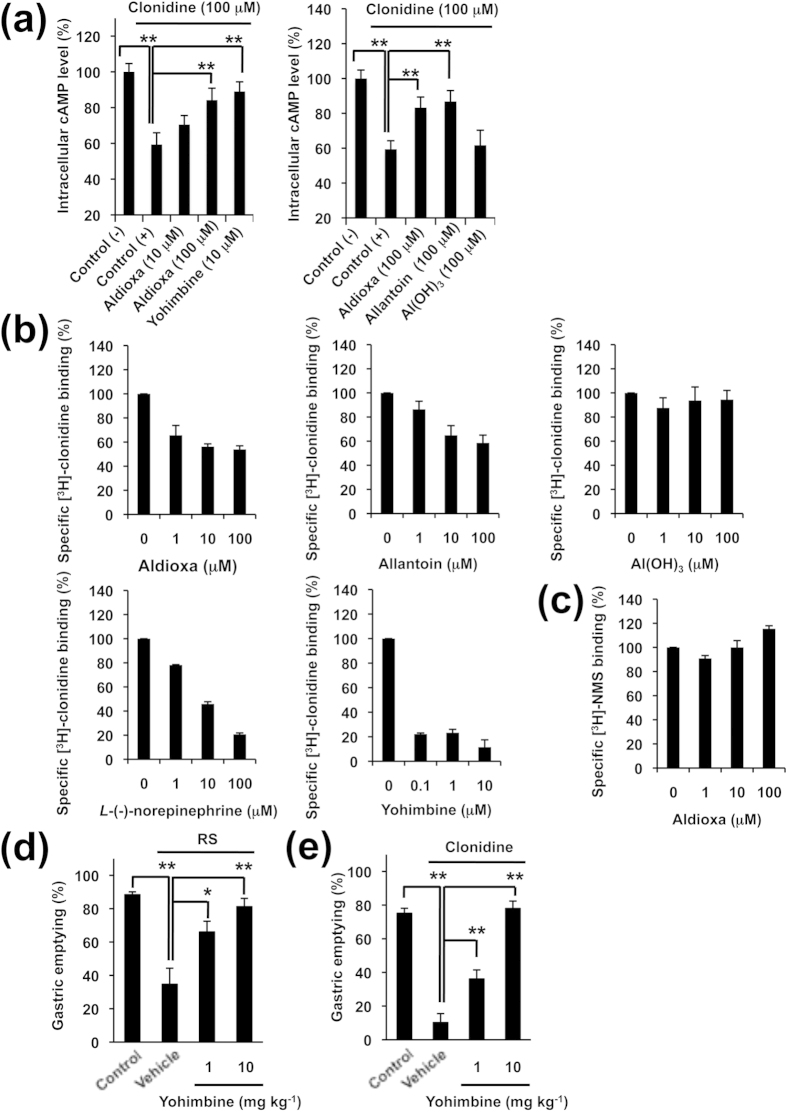
Involvement of α-2 adrenergic receptor in stimulatory effect of aldioxa on gastric emptying. SH-SY5Y cells were incubated with indicated concentration of aldioxa, yohimbine, allantoin or aluminium hydrate (Al(OH)_3_) in the presence of 100 μM clonidine, 5 μM forskolin and 1 mM IBMX for 30 min at 37 °C. Control cells were incubated with 5 μM forskolin and 1 mM IBMX in the presence ( + ) or absence (−) of 100 μM clonidine. Intracellular cAMP levels were determined by ELISA and expressed relative to control (**a**). Membrane fractions prepared from cells expressing human α-2 adrenergic receptor (**b**) or human muscarinic M3 receptor (**c**) were incubated with [^3^H]-clonidine (20 nM) (**b**) or [^3^H]-NMS (2 nM) (**c**), respectively, in the presence of indicated concentrations of aldioxa, allantoin, aluminium hydrate (Al(OH)_3_), *L*-(-)-norepinephrine or yohimbine for 2 h and clonidine- (**b**) or NMS- (**c**) binding was determined by the filter-binding assay. Effect of indicated dose of yohimbine on restraint stress (RS)- (**d**) or clonidine- (**e**) induced delayed gastric emptying was examined using the phenol red method as described in the legends of Figs 1 and 4. Values are mean ± s.e.m. *P < 0.05; **P < 0.01 (Tukey test). Experiments were replicated at least two times.

Next, we tested involvement of the α-2 adrenergic receptor. Since activation of this receptor is coupled with a decrease in the intracellular level of cAMP^31^, we examined the effect of aldioxa on this receptor by monitoring intracellular cAMP levels. As shown in Fig. 6a, treatment of human neuroblastoma (SH-SY5Y) cells with clonidine decreased intracellular cAMP levels in a manner that was suppressed by simultaneous treatment with aldioxa or yohimbine hydrochloride (yohimbine, an α-2 adrenergic receptor antagonist). We also found that allantoin but not aluminium hydrate restored intracellular cAMP levels seen in the presence of clonidine, and that the level of restoration was similar to that observed with aldioxa (Fig. 6b). These results suggest that aldioxa has an antagonizing effect on α-2 adrenergic receptor activation via its allantoin moiety.

To test this idea directly, we examined the binding of aldioxa to the human α-2 adrenergic receptor by carrying out a [^3^H]-clonidine displacement study on this receptor. As shown in Fig. 6b, as for *L*-(-)-norepinephrine (an endogenous ligand for the α-2 adrenergic receptor) and yohimbine, aldioxa inhibited clonidine-binding to the human α-2 adrenergic receptor in a dose-dependent manner, suggesting that aldioxa binds to this receptor. We also found that allantoin but not aluminium hydrate inhibited clonidine binding to the α-2 adrenergic receptor (Fig. 6b). On the other hand, aldioxa did not show an inhibitory effect on *N*-methyl-scopolamine methyl bromide (NMS)-binding to the human muscarinic M3 receptor (Fig. 6c). These results suggest that aldioxa binds specifically to the α-2 adrenergic receptor.

With the above results suggesting that the stimulatory effect of aldioxa on gastric emptying is mediated via its antagonist activity on the α-2 adrenergic receptor, we tested this idea further by examining the effect of yohimbine on delayed gastric emptying. As shown in Fig. 6d, pre-administration of yohimbine suppressed restraint stress-induced delayed gastric emptying. Similar results were observed for clonidine-induced delayed gastric emptying (Fig. 6e).

### Effect of aldioxa on gastric compliance in rats

**Figure 7:**
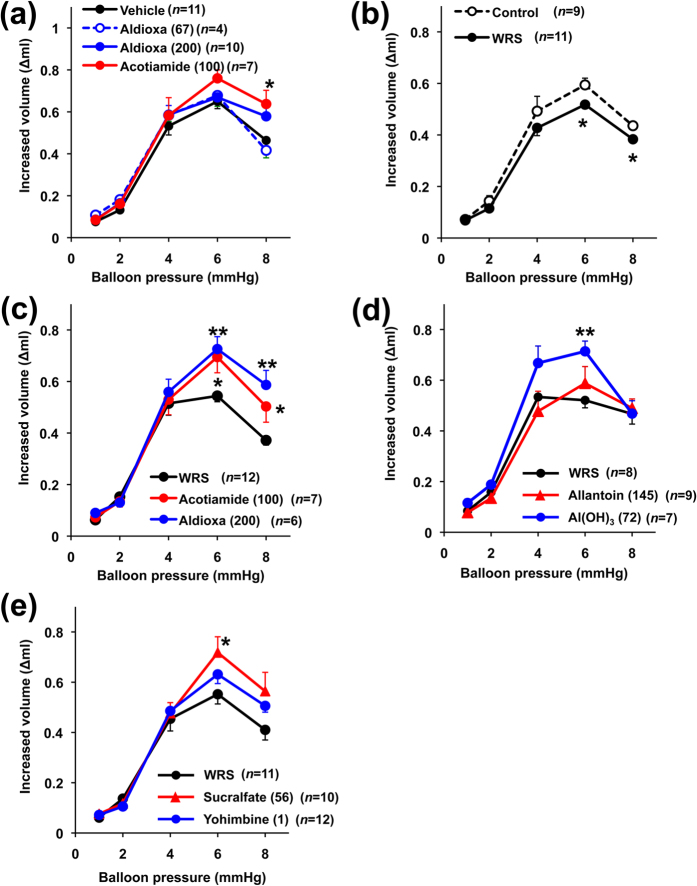
Effect of aldioxa on gastric compliance in rats. Rats fasted for 18 h were orally administered indicated dose of aldioxa (mg kg^−1^) (**a**,**c**), acotiamide (100 mg kg^−1^) (**a**,**c**), allantoin (145 mg kg^−1^) (**d**), aluminium hydrate (Al(OH)_3_) (72 mg kg^−1^) (**d**), yohimbine (1 mg kg^−1^) (**e**), sucralfate (56 mg kg^−1^) (**e**) or vehicle (1% methylcellulose) (**a**,**c**–**e**). Rats were subjected to wrap restraint stress (WRS) for 1 h (**b**–**e**). Gastric compliance was measured one hour after the administration of each drug. Values are mean ± s.e.m. *P < 0.05; **P < 0.01 (Tukey test) (versus vehicle (**a**), versus control (**b**), versus WRS (**c**–**e**). Experiments were replicated at least two times.

We next examined the effect of aldioxa on gastric compliance measured with a barostat apparatus (see Methods). As shown in Fig. 7a, increasing balloon pressure resulted in an increase in balloon volume. The pre-administration of acotiamide to animals potentiated this increase, as described previously in human^18^, showing that acotiamide increases gastric compliance. On the other hand, pre-administration of aldioxa did not significantly affect gastric compliance in control rats (Fig. 7a).

We subsequently examined the effect of aldioxa on gastric compliance in rats subjected to wrap restraint stress. As shown in Fig. 7b, compared to control rats, gastric compliance was decreased in rats subjected to wrap restraint stress. Furthermore, pre-administration of aldioxa or acotiamide increased gastric compliance in these rats (Fig. 7c). These results suggest that administration of aldioxa stimulates gastric compliance under stress conditions.

Finally, we examined the effect of allantoin or aluminium hydrate on gastric compliance. As shown in Fig. 7d, aluminium hydrate but not allantoin increased gastric compliance in rats subjected to wrap restraint stress at a dose equivalent to 200 mg kg^−1^ of aldioxa. We also found that sucralfate (56 mg kg^−1^; a dose equivalent to 72 mg kg^−1^ of aluminium hydrate (or 200 mg kg^−1^ of aldioxa) in terms of the molecular number of contained aluminium) increased gastric compliance in rats subjected to wrap restraint stress. On the other hand, yohimbine did not affect gastric compliance in rats subjected to wrap restraint stress (Fig. 7e). The results in Fig. 7 suggest that aldioxa achieves its stimulatory action on gastric compliance independently of its inhibitory action against the α-2 adrenergic receptor; the effect on gastric compliance is induced by aldioxa’s aluminium hydrate moiety.

## Discussion

A reduced quality of life is a typical consequence of FD in many people suffering from this disease, and this in turn has a significant economic impact on the healthcare system. In spite of this, an effective clinical protocol for the treatment of FD has not been established. Although various types of drugs have been developed (such as 5-HT_4_ agonists and D_2_ antagonists), most of them have drawbacks such as low efficacy and serious adverse effects^8^,^9^. Thus, in order to find new drugs to treat FD, we adopted a strategy of phenotypic screening in animals. Furthermore, to reduce the risk of adverse effects in the clinical setting, we sought to use a drug re-positioning strategy (see introduction).

By screening a library of medicines already used clinically, we found that the oral administration of aldioxa partially suppressed clonidine-induced delayed gastric emptying in mice. Aldioxa was approved in 1971 in Japan and has been used for the prevention and treatment of gastric ulcer and gastritis. We suggest that the stimulatory effect of aldioxa on gastric emptying is independent of its protective effect on the gastric mucosa based on the following: (i) the dose of aldioxa required to affect gastric emptying was much lower than that required to suppress the production of gastric lesions; (ii) other gastroprotective drugs (geranylgeranylacetone and sucralfate) had no effect on delayed gastric emptying.

We found that administration of allantoin but not aluminium hydroxide suppressed the delayed gastric emptying, suggesting that the stimulatory effect of aldioxa on gastric emptying is mediated by its allantoin moiety. This idea is supported by the ineffectiveness on delayed gastric emptying of sucralfate, a drug that also contains aluminium hydroxide.

Aldioxa also suppressed the delayed gastric emptying induced by restraint stress, but did not affect the basal level of gastric emptying in intact mice. Since FD is closely associated with emotional stress, the effectiveness of aldioxa on restraint stress-induced delayed gastric emptying supports it possible beneficial effects on FD patients. On the other hand, since alterations to basal levels of gastric emptying by drugs are related to their adverse effects, the lack of effect of aldioxa on the basal level of gastric emptying in these animals also supports its possible usefulness in FD patients.

It was important to identify the molecular target of aldioxa that mediates its effect on delayed gastric emptying. Since 5-HT_4_ receptor agonists and D_2_ antagonists are used clinically to restore gastric emptying in FD patients, we tested the possibility that aldioxa acts as a 5-HT_4_ receptor agonist or D_2_ antagonist. However, these possibilities were ruled out by observations that aldioxa restored gastric emptying even in the presence of a 5-HT_4_ receptor antagonist but did not affect delayed gastric emptying induced by a D_2_ receptor agonist.

We then tested involvement of the α-2 adrenergic receptor, because aldioxa is effective in overcoming the delayed gastric emptying induced by the α-2 adrenergic receptor agonist clonidine. Treatment of cells with aldioxa or allantoin restored intracellular cAMP levels in the presence of clonidine, with a filter-binding assay revealing that both aldioxa and allantoin inhibit clonidine binding to the human α-2 adrenergic receptor. Furthermore, yohimbine, an α-2 adrenergic receptor antagonist, suppressed restraint stress- or clonidine-induced delayed gastric emptying. All these results support the notion that aldioxa ameliorates delayed gastric emptying through its antagonistic activity on the α-2 adrenergic receptor. Although it is known that α-2 adrenergic receptor antagonism stimulates acetylcholine release from the vague nerve, this receptor has not received much attention as a target of anti-FD drugs^32^. The results of this study, however, suggest that the α-2 adrenergic receptor could be such a target of drugs to improve gastric emptying in FD patients.

We here show that aldioxa suppresses the delayed gastric emptying induced by restraint stress or administration of clonidine. Since restraint stress-induced delayed gastric emptying was reported to involve an adrenergic pathway^7^, it is possible that the restoration by aldioxa would work in conditions only involving an adrenergic pathway. Supporting this notion, aldioxa did not affect delayed gastric emptying induced by a D_2_ receptor agonist. Furthermore, the physiology and anatomy of the rat stomach differ from man^33^. Thus, it is possible that this drug shows ineffectiveness in FD patients. On the other hand, we found that aldioxa at 200 mg kg^−1^ (30 times higher than the clinical dose) shows more potent effect on delayed gastric emptying than cisapride at 10 mg kg^−1^ (60 times higher than the clinical dose), suggesting that the clinical dose of aldioxa shows effectiveness on delayed gastric emptying in human. Therefore, clinical investigations would be required to determine the clinical significance of this effect.

In addition to delayed gastric emptying, impaired gastric accommodation (decrease in gastric compliance) plays an important role in the pathogenesis of FD, particularly PDS^34^. We therefore examined the effect of aldioxa on gastric compliance and found that, in contrast to acotiamide, orally administered aldioxa did not affect gastric compliance in control rats. We did however find that gastric compliance was decreased in rats subjected to wrap restraint stress and that aldioxa increased gastric compliance under such conditions. Concerning the mechanism governing this effect, we found that, in contrast to its stimulatory effect on gastric emptying, aluminium hydroxide but not allantoin or yohimbine restored gastric compliance decreased by wrap restraint stress. These results suggest that aldioxa increases gastric compliance via a mechanism that is independent of inhibitory effect on the α-2 adrenergic receptor, but dependent on its aluminium hydroxide moiety. Supporting this notion, sucralfate (which contains aluminium hydroxide) increased gastric compliance in rats subjected to wrap restraint stress. However, the mechanism how aluminium hydroxide moiety of aldioxa affects gastric compliance has remained unknown. On the other hand, it also should be noted that this is the first demonstration that acotiamide affects gastric accommodation in animals.

Since the present protocol for treating FD has not resulted in satisfactory outcomes on the whole, and a large number of people suffer from this disease, new compounds are currently being developed. However, such an approach involves an enormous financial outlay and the obvious risk of encountering unacceptable side effects during the clinical trial process. Aldioxa might offer a distinct advantage in that its safety has already been clinically demonstrated. Furthermore, because pilot clinical studies are possible for approved medicines, the efficacy (or not) of aldioxa in FD patients can be confirmed without further pre-clinical studies.

In conclusion, the results of this study suggest that aldioxa may be a good candidate drug for treating FD on the basis of its stimulatory effects on both gastric emptying and gastric compliance under conditions where these properties are impaired.

## Methods

### Chemicals and animals

Methylcellulose, carboxymethylcellulose sodium, ethyl carbamate (urethane), clonidine, apomorphine, yohimbine, aldioxa and phenol red were obtained from Wako Pure Chemical Industries (Osaka, Japan). Cisapride, 3-isobutyl-1-methylxanthine (IBMX), forskolin, fetal bovine serum (FBS), allantoin, L-(-)-norepinephrine, GR113808 and mosapride were purchased from Sigma (St. Louis, MO). Itopride and sucralfate were obtained from LKT laboratories Inc. (St Paul, MN). Saline and Racol® were from Otsuka Pharmaceutical Co. Ltd. (Tokyo, Japan). [^13^C]-labeled acetic acid was obtained from Cambridge Isotope Laboratories (Andover, MA). Dulbecco’s modified Eagle’s medium and Ham’s F-12 medium were purchased from Nissui Pharmaceutical Co. Ltd. (Tokyo, Japan). An ELISA kit for cAMP was from ENZO Life Sciences (Farmingdale, NY). Aluminium hydroxide was from Nacalai Tesque Inc. (Kyoto, Japan). Membrane fractions prepared from Chinese hamster ovary (CHO)-K1 cells expressing human α-2A adrenergic receptor or human muscarinic M3 receptor, [benzen ring-^3^H]-clonidine ([^3^H]-clonidine) (61.3 Ci mmol^−1^) and [^3^H]-NMS (85.5 Ci mmol^−1^) were obtained from Perkin-Elmer Life and Analytical Sciences (Boston, MA). Acotiamide and geranylgeranylacetone were gifted from Zeria Pharmaceutical Co. Ltd. (Saitama, Japan) and Eisai Co. Ltd. (Tokyo, Japan), respectively. ICR mice (8-week-old males, 30–35 g) and Sprague-Dawley rats (12-week-old males, 320–370 g) were obtained from Charles River Laboratories Japan (Yokohama, Japan). Animals were housed under conditions of controlled temperature (22–24 °C) and illumination (12 h light cycle) for 1 or 2 weeks before experiments. The experiments and procedures described here were performed in accordance with the Guide for the Care and Use of Laboratory Animals as adopted and promulgated by the National Institutes of Health, and were approved by the Animal Care Committee of Keio University.

### Measurement of gastric emptying using the [^13^C]-labeled acetic acid breath test

Monitoring of the ^13^CO_2_ level in air expired by mice was performed as described previously^23^ with some modifications. The apparatus was composed of a bottle top filter unit (animal chamber; Steritop filter units, Millipore, Billerica, MA), peristaltic pump (Masterflex, Cole-Palmer Instruments Co., Vernon Hills, IL) and a breath-sampling bag (Otsuka Pharmaceutical Co. Ltd., Tokyo, Japan). The animal chamber was wide enough to allow the mouse to turn around. Mice were fasted for 18 h and had free access to water. Racol containing [^13^C]-labeled acetic acid (16 mg acetic acid/kg, 5 ml racol/kg) was then intragastrically administered. Expired air containing ^13^CO_2_ was collected 5, 10, 15, 20, 25, 30, 40, 50, 60 and 90 min after administration of [^13^C]-labeled acetic acid. The ventilation volume was 50 ml min^−1^. The content of ^13^CO_2_ in collected expired air was measured with a POC-one analyzer (Otsuka Electronics Co. Ltd., Osaka, Japan). Values are presented as the difference of ^13^CO_2_/^12^CO_2_ (Δ^13^CO_2_ (%)) between each sample and the standard air. For evaluation of the gastric emptying, we determined the half-time for gastric emptying (*T*_1/2_).

### Measurement of gastric emptying with the phenol red method

Gastric emptying was also monitored by use of the phenol red method as previously described^35^. Mice were fasted for 18 h and had free access to water, following which 1.5% carboxymethylcellulose sodium salt containing 0.05% phenol red (0.5 ml/mouse) was intragastrically administered. Twenty minutes later, mice were sacrificed. After harvesting the stomach, and the gastric content collected and centrifuged at 3,000 rpm for 15 min after treatment with 10 ml of 0.1 M NaHCO_3_. The amount of phenol red in the supernatant was determined based on the absorbance at 558 nm measured using a microplate reader (Multiscan GO, Thermo Scientific, Yokohama, Japan). Phenol red collected from an animal sacrificed immediately after the described administration procedure was used as the standard sample. Gastric emptying (%) was calculated according to the following equation: (1 − amount of phenol red in test sample/amount of phenol red in standard sample) × 100.

### Animal models for delayed gastric emptying, and effect of test compounds on this parameter

Three different animal models of delayed gastric emptying were used, with delayed emptying induced by clonidine, apomorphine or restraint stress.

In the clonidine-induced delayed gastric emptying model, 100 μg kg^−1^ (for phenol red method) or 30 μg kg^−1^ (for [^13^C]-labeled acetic acid breath test) clonidine were subcutaneously administered to mice 5 or 15 min before the administration of phenol red or ^13^C-labeled acetic acid, respectively. In the apomorphine-induced delayed gastric emptying model, mice were subcutaneously administered 5 mg kg^−1^ apomorphine 30 min before the administration of phenol red.

In the restraint stress-induced delayed gastric emptying model, mice were placed individually into a 50 ml Falcon tube (Becton Dickinson, Franklin Lakes, NJ) for 1 h and then [^13^C]-labeled acetic acid or phenol red was administered. These tubes were small enough to restrain a mouse so that it is able to breathe but unable to move freely. Control mice were left to move freely in their cages.

In any of these animal models, each test compound was administered intragastrically with 1% methylcellulose (10 ml kg^−1^) 1 h before the administration of [^13^C]-labeled acetic acid or phenol red.

### Cell culture and determination of the intracellular cAMP level

SH-SY5Y cells were cultured in Dulbecco’s modified Eagle’s medium/Ham’s F-12 medium supplemented with 10% FBS, 100 units/ml penicillin and 100 μg ml^−1^ streptomycin in a humidified atmosphere of 95% air and 5% CO_2_ at 37 °C.

Cells were plated in 24-well plates at a density of 2 × 10^5^ cells/well, incubated for 24 h and then pre-treated with 5 μM forskolin and 1 mM IBMX for 5 min at 37 °C. Cells were then incubated with each test compound in the presence of 100 μM clonidine, 5 μM forskolin and 1 mM IBMX for 30 min at 37 °C. The incubation was stopped by replacing the media with 250 μl ice-cold 0.1N HCl. After 20 min incubation with shaking at room temperature, aliquots were recovered and centrifuged. The amount of cAMP in the supernatant was measured with a cAMP ELISA kit according to the manufacturer’s instructions. Reported values are the means of nine experiments.

### Filter-binding assay

The filter-binding assay was performed as described previously^36^ with some modifications. Membrane fractions prepared from CHO-K1 cells expressing the human α-2 adrenergic receptor or human muscarinic M3 receptor (4 μg protein) were incubated with 20 nM [^3^H]-clonidine or 2 nM [^3^H]-NMS, respectively, at room temperature for 1 h in binding buffer (50 mM Tris-HCl (pH 7.4) and 100 mM NaCl) in the presence of each test compound. The sample was passed through a GF/B filter (F7036, Sigma, St. Louis, MO) that was pre-incubated for 30 min with 1.0% polyethylenimine, and washed four times with ice-cold PBS (pH 7.6). Filters were then dried for 30 min and the radioactivity remaining on the filter was monitored with a Tri-Carb liquid scintillation counter (PerkinElmer Life and Analytical Sciences, Boston, MA). Non-specific binding to the α-2 adrenergic receptor or muscarinic M3 receptor was determined by examining binding in the presence of yohimbine (10 μM) or atropine (2.5 μM), respectively, and specific binding to each receptor was calculated by subtracting the value of non-specific binding. Reported values are the means of three experiments.

### Gastric compliance test

Gastric compliance was examined as described previously^37^ with some modifications. Briefly, rats fasted for 18 h were orally administered each test compound in combination with 1% methylcellulose (2.5 ml/kg). After a 1 h interval (control) or after 1 h of being subjected to wrap restraint stress^38^, rats were anesthetized with urethane (1.5 g kg^−1^). A polyethylene bag (maximum volume, 5 ml; maximum diameter, 3 cm) connected to a pair of polyvinyl tubes was introduced into the stomach, with the tubes exiting via the mouth. Air was injected into the balloon to allow placement of the balloon in the stomach. After a 10 min recovery period, the balloon tubes were connected to a barostat (Barostat Distender IIR, Starmedical, Tokyo, Japan) and the pressure inside the balloon was increased stepwise at 1 min intervals. The balloon volume increased gradually until it reached a plateau. The difference in balloon volume between the initial and the final (plateau) levels was defined as gastric compliance.

### Gastric damage assay

The gastric ulcerogenic response was examined as described previously^39^,^40^ with some modifications. Mice fasted for 18 h were orally pre-administered each test compound and after 1 h, orally administered indomethacin (20 mg kg^−1^). After 8 h, the animals were sacrificed, their stomachs were removed, and the gastric mucosal lesion area was measured by an observer unaware of the treatment the animals had received. Calculation of the scores involved measuring the area of all the lesions (expressed in square millimetres) and summing the values to give an overall lesion index.

### Statistical analysis

Each value is expressed as the mean ± S.E.M. Two-way ANOVA followed by the Tukey test or the Student’s *t*-test for unpaired results was used to evaluate differences between more than two groups or between two groups, respectively. Differences were considered to be significant for values of P < 0.05.

## Additional Information

**How to cite this article**: Asano, T. *et al.* Aldioxa improves delayed gastric emptying and impaired gastric compliance, pathophysiologic mechanisms of functional dyspepsia. *Sci. Rep.* **5**, 17519; doi: 10.1038/srep17519 (2015).
